# Treating patients with fibromyalgia in primary care settings under routine medical practice: a claim database cost and burden of illness study

**DOI:** 10.1186/ar2673

**Published:** 2009-04-14

**Authors:** Antoni Sicras-Mainar, Javier Rejas, Ruth Navarro, Milagrosa Blanca, Ángela Morcillo, Raquel Larios, Soledad Velasco, Carme Villarroya

**Affiliations:** 1 Directorate of Planning, Badalona Serveis Assistencials, C. Gaietà Soler, 6-8 entresuelo, Badalona, Barcelona, 08911, Spain; 2 Department of Health Outcomes Research, Medical Unit, Pfizer España, Avda de Europa 20B, Parque Empresarial la Moraleja, Alcobendas, Madrid, 28108, Spain; 3 Department of Psychiatry, Badalona Serveis Assistencials, C. Gaietà Soler, 6-8 entresuelo, Badalona, Barcelona, 08911, Spain; 4 Department of Family Medicine, Badalona Serveis Assistencials, C. Gaietà Soler, 6-8 entresuelo, Badalona, Barcelona, 08911, Spain

## Abstract

**Introduction:**

The objective of this study was to analyze health care and non-health care resource utilization under routine medical practice in a primary care setting claims database and to estimate the incremental average cost per patient per year of fibromyalgia syndrome (FMS) compared with a reference population.

**Methods:**

A 12-month cross-sectional and retrospective study was completed using computerized medical records from a health provider database. Analyses were conducted from the perspective of the provider and from the viewpoint of society. Health care and non-health care resource utilization included drugs, complementary tests, all types of medical visits, referrals, hospitalizations, sick leave, and early retirement because of disability due to FMS. Patients with a diagnosis of FMS in accordance with ICD-10 (International Statistical Classification of Diseases and Related Health Problems, 10th revision) criteria were included in the analysis if they had at least one claim for FMS during the 12 months prior to the end of May 2007. A non-FMS comparison group was also created with the remaining subjects.

**Results:**

Of the 63,526 patients recruited for the study, 1,081 (1.7%) (96.7% of whom were women, 54.2 [10.1] years old) met the criteria for FMS. After an adjustment for age and gender, FMS subjects used significantly more health care resources than the reference population and had more sick leave and the percentage of subjects with premature retirement was also significantly higher (*P *< 0.001 in all cases). As a result, FMS subjects showed an incremental adjusted per-patient per-year total cost of €5,010 (95% confidence interval [CI] 3,494 to 6,076, +153%, *P *< 0.001) on average compared with non-FMS subjects. Significantly higher differences were observed in both health care and non-health care adjusted costs: €614 (404 to 823, +66%) and €4,394 (3,373 to 5,420, +189%), respectively (*P *< 0.001 in both cases). Annual drug expenditure per patient on average was considerably higher in FMS patients, €230 (124 to 335, +64%, *P *< 0.001), than the reference group.

**Conclusions:**

Under routine medical practice, patients with FMS were associated with considerably higher annual total costs in the primary care setting compared with the reference population.

## Introduction

Fibromyalgia syndrome (FMS) is characterized by widespread pain, tenderness, and fatigue and is typically difficult to diagnose [1]. In 1990, the American College of Rheumatology (ACR) published diagnostic criteria for FMS – namely, widespread pain (both sides of the body, above and below the waist, and in the cervical spine, anterior chest, thoracic spine, or lower back) and pain upon digital palpation in at least 11 of 18 specified tender point sites [2] – although it was not officially recognized as an illness by the World Health Organization until 1992 [3]. FMS is a widespread disorder of unknown etiology which affects an estimated 1% to 4% of the general population [4]. It may occur in 2.1% to 5.7% of the general adult population, comprising 10% to 20% of rheumatologic consultations and 5% to 8% of primary care (PC) consultations and being the most frequent cause of general and chronic skeletal muscular pain [5-7]. Women are about nine times more likely than men to develop FMS [1].

The symptoms of FMS can be prolonged and debilitating. It negatively affects the lives of patients, the people around them, and the environment in which they live. It is one of the rheumatic illnesses with the greatest impact on patient quality of life, having negative consequences on physical capability, intellectual activity, emotional condition, personal relationships, professional career, and mental health to the extent that the patient requires multiple intervention strategies [8-10]. In recent years, fibromyalgia (FM) has acquired greater significance and has become a first-order public health problem. There are several reasons to justify this situation: (a) its high level of prevalence in the general adult population, (b) insufficient knowledge of its cause and the mechanisms that produce it (decrease of the nociceptive perception threshold), (c) absence of a curative treatment, and (d) dissatisfaction of patients and professionals with current therapeutic approaches [7-9]. Given the chronicity of the symptomatology and the disability that it often produces, it is associated with elevated levels of health care and non-health care resources, often stemming from work absenteeism [9].

Available evidence on the cost of FMS to society has been scant up to now, and information on the direct and indirect costs and utilization of health care resources comes primarily from the US, Canada, and The Netherlands [11-15]. In these countries, the direct health care costs are considerable, and the indirect costs, arising from employment absenteeism and disability pensions, are double those of the general working population. Total annual expenses for a patient with FMS entail more than twice the expenses incurred for a patient with ankylosing spondylitis and are similar to those of a patient with chronic lumbalgia [11-16].

There are substantial limitations to the existing research. Several studies were conducted well over a decade ago, and many of the more recent ones have other shortcomings, including small sample size and/or choice of reference group. Moreover, many of these studies are based on questionnaire data, which may not necessarily reflect actual patterns of utilization because of problems with patient recall and/or comprehensiveness of questionnaire content. A large study has been conducted in the US using a large health insurance claims database and for the year 2005 showed an annual health care cost per patient with FMS of $9,573, which is three times higher than that of the reference group [14]. However, patterns of medical care in the US may differ widely from those in the European context. We have not been able to identify any cost studies for FMS in the Spanish health system. The objective of this study was to analyze the use of health care and non-health care resources in a primary health care setting and the costs arising from the treatment of patients with FM under usual medical practice conditions recorded in a Spanish claims database. Information on the economic impact of FMS will be useful to clinicians, payers, and researchers.

## Materials and methods

### Study design and data collection

A cross-sectional and multicenter study was conducted from a retrospective review of medical outpatient records. The study population consisted of men and women from five renovated PC centers (Apenins-Montigalà, Morera-Pomar, Montgat-Tiana, Nova Lloreda, and La Riera) that are managed by a health management organization (Badalona Serveis Assistencials S.A. [BSA], Barcelona, Spain) and that cover a population of 110,440 inhabitants, 16.5% of whom are over 64 years of age. The assigned population is primarily urban. The organization is public with a private services supply and is managed according to a business model. The corporation staff, training policy, organization model, and services portfolio are similar to those of most PC centers in Catalonia (Spain), with a decentralized management model and with integrated structural services. This study is based on data obtained from an administrative medical database; consequently, it was not necessary to be approved by an ethics committee.

We analyzed men and women (older than 18 years of age) who were included in the database (n = 63,526). FMS patients, with a code in accordance with ICD-10v10 (International Statistical Classification of Diseases and Related Health Problems, 10th revision) (code M79.7) criteria for this disorder, were included in the analysis if they had at least one claim for FMS between 1 May 2006 and 30 April 2007. Subjects who did not have a claim for FMS were included in a reference group. Subjects referred to other PC centers, belonging to other geographic areas, visiting integrated specialists, or having serious psychiatric illnesses were excluded from the study. The clinical diagnosis of FMS was based on the presence of chronic and generalized bone and muscular pain, according to the ACR classification criteria established in 1990 [2]. FMS is defined by a history of at least 3 months of generalized and continuous pain on both sides of the body, above and below the waist, and axial skeleton, cervical, or front chest pain. Furthermore, there should be pain to touch in at least 11 out of the following 18 symmetric points: occipital, low cervical, trapezium, supraspinal, second intercostal space in the chondrocostal joint, epicondyle, gluteal, greater trochanter, and knee.

The study was performed in two phases. In the first, information on the primary sociodemographic and clinical (comorbidity) variables and the use of health care and non-health care resources (days of leave from work, subjects with permanent disability) related to the FMS was obtained from the database, omitting any data that could identify the patient. Information on resources used by patients as a consequence of their illness which were not financed by the National Health System such as special diets or non-pharmacological treatments (such as massages or acupuncture) was not gathered as it was not included in the database. In the second phase, telephone interviews were conducted of a sample of FMS patients selected at random from the database to evaluate self-perceived health and well-being. Interviews were conducted by members of the research team. The surveys averaged 40 minutes in duration and were conducted in the months of May and June of 2007, immediately after the data on the use of health care and non-health care resources were collected from the database. Prior to the interview, the selected patients were contacted to inform them about the study, obtain their consent for participation, and provide a verbal guarantee of confidentiality. Interviewers were previously subjected to training on the instruments of self-perceived health which had to be administered (see 'Patient-reported outcome instruments used in the study'). Selection of individuals was made using probability techniques (simple random sample stratified by age and gender), and sample size was calculated by adopting the following parameters: confidence level of 95%, bilateral test, infinite populations, precision of 2%, and anticipated prevalence of the illness of 2.2%. The total number of subjects to be interviewed was 212. Persons with a physical or psychiatric disability that limited them from responding to a telephone questionnaire, persons with incorrect phone numbers, subjects who were not located after three calls made on different days, and those who declined to participate were considered losses to the study.

The primary recorded data were age (continuous and by ranges), gender, and personal history (comorbidities) obtained from the PC International Classification (CIAP-2) [17], 7th component of diseases and health problems, consisting of blood hypertension (K86, K87), dyslipidemia (T93), diabetes mellitus (T90, all types), active smoking (P17), alcoholism (P15), obesity (T82), ischemic cardiopathy (K74, cardiac ischemia with angina; K75, acute myocardial infarction; K76, coronary ischemia), cerebrovascular events (including stroke and transient ischemic attack), presence of a cardiovascular event, chronic obstructive pulmonary disease (R95, chronic obstruction of airflow), bronchial asthma (R96), depressive syndrome (P70), failure of all types (heart, liver, and renal), neuropathy, malignancy, and dementia. Morbidity burden (patient's severity) and the number of health problems attended per patient per year were assessed using the Charlson index [18] and the resource utilization band (RUB) method [19].

### Health and non-health care resources and cost estimation

Health resource utilization obtained from center records consisted of visits or appointments conducted at the PC center, referrals to reference specialists, requests for complementary support tests, emergency room visits, hospitalizations, and drug prescriptions financed by the National Health Service. Non-health care resource utilization consisted of workdays lost in the active population and early retirement (<65 years old) because of permanent disability due to FMS. The cost system design was defined by taking into account the characteristics of the organization and the level of development of available information systems. The unit of assistance used as the base for final calculation was the cost per patient assisted during the study period. Fixed costs (with imputation criteria) and variable costs were considered in accordance with their dependence on the volume of activity. Costs related to staff (salaries), consumer goods, and a set of expenses related to external services, in accordance with the General Accountability Plan for Health Care Centers, were considered fixed costs (structural), and those associated with diagnostic, therapeutic, or referral requests performed by staff at the center were considered variable costs. Economic assessments were generated for (a) complementary tests, including laboratory tests (mean expenses per application), conventional radiology (fee per each test requested), and support tests (fee per each test requested); (b) ordinary or urgent referrals to reference specialist doctors or to hospitals (referral adapted fee); (c) prescriptions (acute, chronic, or requested medical prescriptions; market price per container), and (d) workdays lost (professional average salary) and information regarding early retirement (before 65 years) because of permanent disability due to FMS. The fees used were obtained from analytical accountability studies conducted by the organization or from CatSalut established prices [20]. The mean cost per visit was obtained from average fixed costs, and a final direct distribution was made for each patient assisted during the study period.

Indirect costs were calculated according to human capital methodology [21,22]. Two main components of these costs were calculated. First, workdays lost due to sick leave in the active population, as a result of FMS, were calculated as the sum of the yearly number of workdays lost (recorded in the BSA database during the study period) multiplied by daily average salary in active subjects. Second, we added the cost to society for those patients with early retirement prior to 65 years of age (permanent disability from usual working activity) due to FMS. These costs were calculated as the sum of the average salary for the calendar year (which is considered to be an opportunity cost) plus the pension received from Social Security because of the permanent disability from performing usual tasks, resulting in early retirement prior to 65 years of age. The annual average professional salary in the year 2006 was €18,714 and the pension received because of a permanent disability from Social Security was €1,906 per year per subject. Thus, the total cost per patient (Cp) was Cp = (mean cost per visit × number of visits [average fixed costs]) + variable costs + indirect costs. All costs were expressed in euros for the year 2007 and are shown as mean cost per patient per year.

### Patient-reported outcome instruments used in the study

Self-perceived health included self-assessment of pain intensity and impact on various facets of the patient's life, health condition, and quality of life as it relates to health. Validated Spanish versions of the following health instruments were administered: (a) Fibromyalgia Impact Questionnaire (FIQ) [23,24], (b) the modified Brief Pain Inventory (BPI)-Short Form [25,26], and (c) the five-item questionnaire on European quality of life (EQ-5D) [27,28]. The FIQ was developed in an attempt to capture the total spectrum of problems related to FM and responses to therapy. The FIQ is a self-administered instrument that takes approximately 3 to 5 minutes to complete. It is scored in such a way that a higher score indicates a greater impact of the syndrome on the person. Each of the 10 items has a maximum possible score of 10. Thus, the maximum possible score is 100. The first item consists of 11 questions that make up a physical function scale. The 11 questions are scored and added to yield one physical impairment score. Each item is rated on a 4-point Likert-type scale. Items 2 and 3 have a 0 to 7 score range and then require standardization to a 0 to 10 scale. Items 4 through 10 are scored in a 0 to 10 score range and they do not require standardization. Finally, the FIQ total store is calculated by adding the scores of all 10 items [24]. The BPI is an instrument developed for use in epidemiological studies and clinical trials to evaluate the effectiveness of pain treatment. It consists of two dimensions: pain intensity (four items) and interference with activities (seven items). Each one of the items is scored using a numeric rating scale from 0 (absence of pain/interference with daily life) to 10 (worst pain imaginable/maximum impact on daily life). Scores for each subscale are obtained using the sums of the partial scores from the corresponding items divided by the number of items in each subscale, although a direct interpretation of each one of the items individually can be made [26]. The pain severity subscale can be interpreted in a categorized manner in three levels of intensity: mild (<4), moderate (≥ 4 to <7), and severe (≥ 7) [29]. A score greater than or equal to 5 on the interference subscale is considered to indicate the existence of pain interference in the patient's daily activities (this interpretation is also valid for each one of the items on the subscale) [30]. The EQ-5D was designed to assess the patient's perceived health status. This is a five-item generic measure of health status to assess the level of abnormality on five dimensions: movement, self-care, daily life activities, pain/discomfort, and anxiety/depression. Scores of these five items may be used to calculate a utility index, ranging from -0.6 to 1.0, with higher scores representing better health status. The instrument also includes a 20-cm visual analogue scale (EQ-5D VAS) ranging from 0 (the worst imaginable health status) to 100 (the best imaginable health status) [27,28].

### Statistical analysis

As a step prior to analysis, in particular to the source of information pertaining to computerized clinical records (Oficina Médica Informatizada de Atención Primaria Windows version, STACKS, Barcelona, Spain), data were carefully reviewed to study the distribution of frequencies and to check possible recording or codifying errors. The quality of computer-obtained data was considered adequate, and legal confidentiality requirements for recording were maintained as previously mentioned. A descriptive univariate statistical analysis was conducted, and the Kolmogorov-Smirnov test was used to check normality of the distribution. For the bivariate analysis, Student *t *tests and chi-square tests were used. A bivariate logistic regression analysis and analysis of covariance (ANCOVA) were conducted to adjust FMS findings (comorbidities, premature retirements, type of treatment, and so on) by age and gender. Costs between reference group and FMS patients were compared, in accordance with the recommendations of Thompson and Barber [31], using an ANCOVA with gender and age as covariates (with Bonferroni corrections for multiple-pair comparisons).

Analyses of correlation, with Pearson product-moment coefficient calculation, were carried out to explore the possible relationship between patient-reported outcome questionnaire responses and cost of disease (total, health care, and indirect costs). Linear regression analyses were carried out between costs and the interference of pain with daily activities (BPI-I subscale with patients grouped according to the degree of interference in 10 categories from 0–1 to 9–10) and disease impact (FIQ total scoring expressed in decile intervals) in which the coefficient of correlations was at least 0.2 to explore the ability of such instruments to relate disease impact/interference with costs. The SPSS/WIN program (version 14) (SPSS Inc., Chicago, IL, USA) was used, and a *P *value of less than 0.05 was considered statistically significant.

## Results

### Demographics and clinical characteristics

From the 110,440 subjects assigned to the five centers initially selected, 80,775 attended PC settings during the study period, with an intensity of use of 73.1% and a frequency of 4.7 visits per 100 inhabitants per year. Ultimately, 63,526 were recruited for the study. During the study period, 304,075 health problems and 494,122 PC visits were recorded; on average, 0.61 health problems were attended per visit per year. One point seven percent (n = 1,081; 95% confidence interval 0.6% to 2.2%) of patients had a diagnosis of FMS according to ICD-10 criteria. General characteristics, sociodemographics, and principal comorbidities of patients studied are presented in Table 1. The mean age of FMS subjects was slightly but significantly higher compared with the reference group (54.2 [10.1] versus 49.1 [17.9], *P *< 0.001), whereas the percentage of women was, as expected, significantly higher (96.7% versus 53.5%, *P *< 0.001). FMS patients showed significant and higher prevalence of a variety of common comorbidities such as psychiatric disorders (major depression, anxiety, and so on), neurological diseases (migraine), pain, and digestive tract diseases (gasroesophageal reflux disease [GERD], gastritis, and so on), with significantly higher values of morbidity burden (RUB and Charlson index). Of particular interest were migraines, major depressive disorders, irritable bowel syndrome, GERD, and pain that had gender- and age-adjusted odds ratios above 2 compared with the reference population. FMS patients also showed significantly higher values of total cholesterol, low-density lipoprotein cholesterol, and triglycerides compared with subjects in the control group (Table 2).

**Table 1:** Demographic and clinical characteristics of study subjects

Demographics	FMS patients n = 1,081	Reference group n = 62,445	Statistics^a^	*P *value
Age in years, mean (SD)	54.2 (10.1)	49.1 (17.9)	16.29	<0.001
18–44	15.6	45.1	3.09	<0.001
45–64	71.2	32.9		
65–84	13.0	19.8		
≥ 85	0.1	2.2		
Gender, percentage of females	96.7	53.5	796.02	<0.001
Pensioners, percentage	42.8	31.2	1.46 (1.26–1.70)	<0.001
BMI in kg/m^2^, mean (SD)	28.6 (5.3)	27.4 (5.1)	7.06	<0,001
Smoking, percentage	18.2	21.0	1.35 (1.15–1.58)	<0.001
Alcoholism, percentage	0.7	1.7	1.55 (0.76–3.16)	0.229

Main comorbidities^b^				

Blood hypertension	27.8	21.8	1.37 (1.19–1.59)	<0.001
Dyslipemia	37.0	23.3	1.94 (1.71–2.21)	<0.001
Diabetes mellitus	8.4	9.1	1.09 (0.87–1.36)	0.449
Obesity/weight gain (BMI > 27 kg/m^2^)	41.1	31.7	1.34 (1.18–1.53)	<0.001
Coronary heart disease	1.8	3.6	1.02 (0.64–1.63)	0.924
Stroke	3.3	4.1	1.38 (0.98–1.95)	0.064
Peripheral vascular disease	4.8	6.9	1.25 (0.94–1.67)	0.131
COPD	0.9	2.8	1.06 (0.56–7.17)	0.855
Asthma	6.1	4.0	1.36 (1.06–1.76)	0.018
Any neurological disorders	0.4	0.5	0.77 (0.28–2.07)	0.601
Dementia	0.4	0.9	0.88 (0.32–2.38)	0.794
Migraine	12.6	3.6	2.41 (1.99–2.92)	<0.001
Major depressive disorders	40.2	10.7	3.85 (3.39–4.37)	<0.001
Anxiety disorders	31.3	19.7	1.24 (1.09–1.42)	0.001
Any disease of the digestive system	70.3	54.0	1.44 (1.24–1.67)	<0.001
Irritable bowel syndrome	5.1	1.2	2.56 (1.91–3.44)	<0.001
GERD	14.2	5.0	2.14 (1.77–2.59)	<0.001
Gastritis	22.4	11.6	1.51 (1.29–1.78)	<0.001
Pain	20.5	5.5	2.94 (2.51–3.43)	<0.001
Neoplasm	4.3	3.5	1.23 (0.91–1.66)	0.187
Morbidity burden				
Charlson index, mean (SD)	0.29 (0.62)	0.26 (0.58)	1.71	0.089
RUB index				
Low	9.8	27.2	354.8	<0.001
Moderate	76.2	52.3		
High	9.7	5.7		
Very high	0.8	0.5		
Healthy	3.4	14.3		

^a^Values of *t *or chi-square for age, body mass index (BMI), gender, age group, and morbidity burden comparisons; other values are odds ratio adjusted for age and gender with 95% confidence intervals in parentheses. ^b^Unless otherwise indicated, all values are percentages. COPD, chronic obstructive pulmonary disease; FMS, fibromyalgia syndrome; GERD, gastroesophageal reflux disease; RUB, resource utilization band; SD, standard deviation.

**Table 2:** Analytical parameters by study group

Characteristic	FMS patients^a^ n = 1,081	Reference group^a^ n = 62,445	Adjusted differences and *P *value^b^
Systolic blood pressure, mm Hg	124.6 (17.4)	126.5 (17.5)	1.6 (-1.1–4.3); *P *= 0.251
Diastolic blood pressure, mm Hg	76.1 (9.9)	75.4 (10.0)	0.8 (-0.9–2.5); *P *= 0.337
Glucose, mg/dL	95.8 (23.3)	96.9 (27.1)	3.5 (-0.9–7.7); *P *= 0.116
Hemoglobin A1c, percentage	6.0 (1.5)	6.4 (1.5)	0.4 (-0.8–0.1); *P *= 0.115
Triglycerides, mg/dL	126.5 (120.6)	119.2 (84.1)	28.5 (12.8–44.1); *P *< 0.001
Total cholesterol, mg/dL	213.9 (38.4)	200.9 (40.9)	14.4 (7.7–21.1); *P *< 0.001
HDL-C, mg/dL	61.1 (17.3)	56.9 (17.5)	0.2 (-3.0–3.3); *P *= 0.913
LDL-C, mg/dL	130.3 (35.7)	123.0 (35.9)	14.1 (7.3–21.0); *P *< 0.001

^a^Values are presented as mean (standard deviation). ^b^Differences adjusted for age and gender. FMS, fibromyalgia syndrome; HDL-C, high-density lipoprotein cholesterol; LDL-C, low-density lipoprotein cholesterol.

### Resource utilization and costs

As a consequence of the higher morbidity burden and prevalence of comorbidities, the FMS group was associated with higher use of pain-related medications such as antidepressants, long-acting opioids, analgesics, and muscle relaxants (Table 3). Fifty-two point four percent of patients with FMS used benzodizepines versus 19.6% in the reference group (*P *< 0.001), 74.7% of subjects used nonsteroidal anti-inflammatory drugs versus 39.3% (*P *< 0.001), 25.3% used muscle relaxants versus 6.5% (*P *< 0.001), 30.5% used long-acting opioids versus 3.8% (*P *< 0.001), and 63.5% used other types of analgesics versus 33.3% (*P *< 0.001) in the general population. On average, after adjustments for age and gender, subjects with FMS were treated, in one year, with 1.96 more drugs than the reference population (Table 3). In addition, patients with FMS used significantly more health care resources (medical visits, referrals to specialists, and supplementary tests), except for hospital stays, than the reference group (Table 4). It is particularly interesting to note that patients with FMS made, on average, six more yearly medical visits than the reference population: five to the PC physician and one other visit to a specialist or to emergency services (*P *< 0.001 in all cases) (Table 4). Greater use of health care resources was accompanied by a higher average of workdays missed (20.9 versus 8.0 days, *P *< 0.001) and a greater number of subjects who receive a pension from Social Security due to permanent disability before the theoretical retirement age (29.9% versus 9.5%, *P *< 0.001) (Table 4).

**Table 3:** Distribution of study subjects according to principal related medications

Pain-related medication	FM patients n = 1,081	Reference group n = 62,445	Odds ratio (95% CI)	*P *value
Antiepileptics	16.0	4.3	1.18 (0.97–1.44)	0.102
Benzodiazepines	52.4	19.6	1.47 (1.27–1.70)	<0.001
Sedatives and hypnotics	12.8	5.3	0.99 (0.81–1.22)	0.949
Corticosteroids	6.4	3.2	0.97 (0.73–1.29)	0.851
COX-2 inhibitors	0.6	0.2	0.81 (0.30–2.17)	0.673
NSAIDs	74.7	39.3	1.86 (1.59–2.17)	<0.001
Muscle relaxants	25.3	6.5	2.20 (1.88–2.59)	<0.001
Antidepressants				
TCAs	22.2	1.8	5.46 (4.56–6.53)	<0.001
MAOIs	0.0	0.0	0.00 (0.00–0.00)	0.999
SSRIs	36.3	9.8	2.08 (1.79–2.41)	<0.001
Other antidepressants	16.3	2.2	2.90 (2.37–3.55)	<0.001
Opioids				
Short-acting opioids	0.1	0.1	0.20 (0.03–1.51)	0.118
Long-acting opioids	30.5	3.8	3.56 (3.02–4.21)	<0.001
Antimigraine drugs				
Triptans	3.5	0.9	1.05 (0.71–1.57)	0.802
Other antimigraine drugs	2.5	0.5	1.58 (1.00–2.49)	0.048
Other analgesics	63.5	33.3	1.54 (1.33–1.78)	<0.001
Miscellaneous	11.6	4.4	0.94 (0.75–1.16)	0.549
Medications per year, mean (SD)	3.7 (2.0)	1.4 (1.5)	1.96 (1.73–2.18)^a^	<0.001

Unless otherwise indicated, all values are percentages. ^a^Value is the mean difference (95% CI) between groups adjusted by age and gender. CI, confidence interval; COX-2, cyclooxygenase-2; FM, fibromyalgia; MAOI, monoamine oxidase inhibitor; NSAID, nonsteroidal anti-inflammatory drug; SD, standard deviation; SSRI, selective serotonin reuptake inhibitor; TCA, tricyclic antidepressant.

**Table 4:** Annual use and cost of health care and non-health care resources among study subjects by group

Resource/type of cost	FMS patients n = 1,081	Reference group n = 62,445	Adjusted differences and/or odds ratio (95% CI) and *P *value^a^
Mean annual use of health care and non-health care resources			

GP office visits	13.5 (10.1)	7.7 (8.2)	5.0 (3.8–6.3); *P *< 0.001
Complementary tests^b^	0.4 (0.7)	0.2 (0.5)	0.3 (0.2–0.4); *P *< 0.001
Referrals	1.5 (1.6)	0.8 (1.1)	0.7 (0.5–0.8); *P *< 0.001
Emergency room visits	0.6 (1.1)	0.4 (0.8)	0.3 (0.2–0.4); *P *< 0.001
Hospitalizations, stays	1.48 (0.79)	1.47 (1.07)	0.44 (-0.29–1.17); *P *= 0.239
Sick leave, days	20.9 (57.2)	8.0 (30.7)	23.5 (18.3–28.7); *P *< 0.001
Premature retirement, %^c^	29.9	9.5	3.58 (3.13–4.10); *P *< 0.001^d^

Mean annualized costs in euros, total and disaggregated in components			

Health care costs	1,677.3 (1,467.4)	934.8 (1,390.2)	613.6 (403.7–823.4); *P *< 0.001
Physician visits^b^	424.0 (304.7)	233.5 (236.1)	171.7 (134.7–208.7); *P *< 0.001
Complementary tests	275.9 (282.8)	126.6 (198.6)	108.8 (77.2–140.3); *P *< 0.001
Hospitalizations	268.6 (760.1)	213.9 (783.9)	101.7 (-27.1–230.5); *P *= 0.122
Drugs	706.5 (840.8)	359.7 (710.2)	229.8 (124.1–335.4); *P *< 0.001
Non-health care costs	6,977.0 (9,256.6)	2,330.5 (6,100.9)	4,396.5 (3,373.2–5,419.8); *P *< 0.001
Sick leave	815.8 (2,565.3)	376.0 (1,486.1)	836.8 (587.1–1,086.5); *P *< 0.001
Early retirement^c^	6,161.2 (9,442.8)	1,954.5 (6,040.1)	3,559.7 (2,547.8–4,571.6); *P *< 0.001
Total cost	8,654.3 (9,645.7)	3,265.3 (6,421.5)	5,010.1 (3,494.0–6,076.2); *P *< 0.001

Values are presented as mean (standard deviation). ^a^Differences are adjusted for age and gender. ^b^Includes all types of medical visits, referrals, and emergency room visits. ^c^Permanent disability for work as a consequence of fibromyalgia syndrome (FMS) before 65 years of age in active population only. ^d^Adjusted odds ratio for age and gender. CI, confidence interval; GP, general practitioner.

Age was positively and significantly correlated to health care costs (*r *= 0.280, *P *< 0.001), whereas a weaker correlation, though significant, was shown with indirect costs (*r *= 0.072, *P *= 0.018) and total costs (*r *= 0.112, *P *< 0.001). This is explained by the fact that costs of lost earnings are not calculated after the theoretical retirement age, only health care costs.

The greater use of health care resources and absenteeism/employment disability in FMS patients is accompanied by significantly higher costs, in both the direct (except for hospitalization costs) and the indirect (sick leave and early retirement) costs. Once adjustments for age and gender were made, FMS subjects incurred €614 more in average annual health care costs (*P *< 0.001) (Table 4) and €4,397 more in the component of indirect costs (*P *< 0.001) in comparison with the reference group, totaling an extra annual average cost per patient of €5,010 (*P *< 0.001) (Table 4).

### Self-perceived health and costs

A second analysis assessed the relationship of total costs, direct and indirect, to the impact of FMS on self-perceived health, as determined using the FIQ, BPI, and EQ-5D scales mentioned above. The measures were administered to a subsample of 200 patients selected at random from among the 1,081 subjects with FMS included in the database. This patient population did not differ from the overall patient sample with FMS in the proportion of women (97.5% versus 96.5%, respectively; chi-square = 0.26, *P *= 0.612), pensioners (38.0% versus 43.9%, respectively; chi-square = 2.06, *P *= 0.152), or comorbidities according to the Charlson index (chi-square = 2.16, *P *= 0.142). Slightly significant differences in the mean age were noted (53.0 [8.5] versus 54.5 [10.4] years old, respectively; *t *= 1.92, *P *= 0.06), and as such, the sample to whom the measures were administered was considered to be comparable to and representative of the population of FMS patients in the BSA database. Persons with a physical or psychiatric disability that limited them from responding to a telephone questionnaire (n = 2), those with incorrect phone numbers (n = 3), subjects who were not located after three calls made on different days (n = 5), and those who declined to participate were considered losses to this analysis. In the end, information from the health questionnaires was obtained from a total of 200 patients. The mean score (standard deviation) in the FIQ for this subsample was 71.7 (16.9) points, with 6.9 (1.7) and 6.8 (2.1), respectively, as mean scores on the subscales of pain severity and interference on the BPI questionnaire. More than three quarters of the patients had pain interference with their daily activities (score of at least 5 on the pain interference subscale of the BPI), and 57% stated that the condition of their health had worsened with respect to the previous year (EQ-5D).

**Figure 1:**
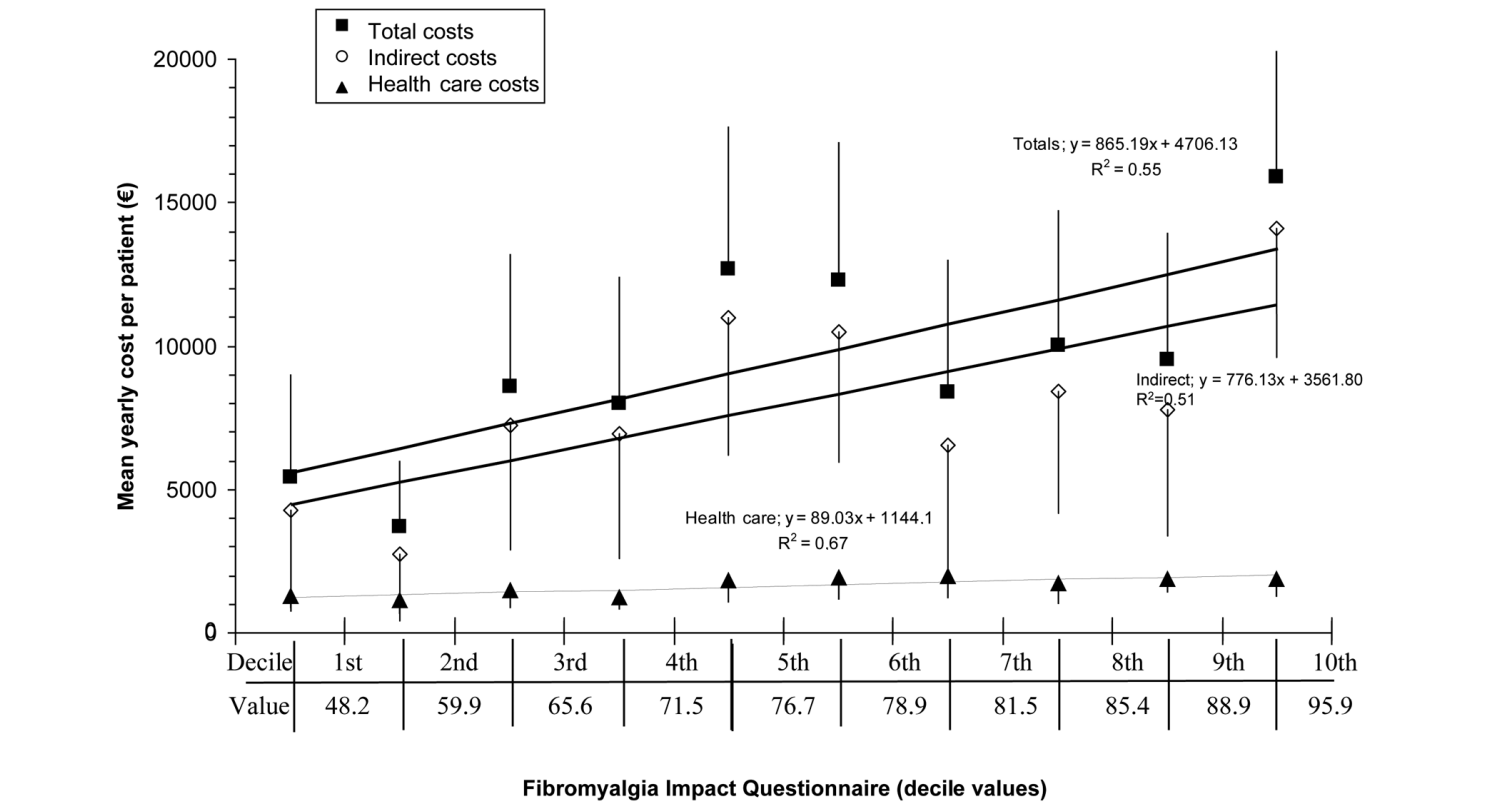
Ability of the impact of the disease (FIQ) to explain annual mean costs. The range of the FIQ is from 0 to 100. Explanatory ability assessed by linear regression models of decile values of the FIQ and corresponding costs at the decile interval of the instrument are shown. Cost values are expressed as inter-decile interval mean ± 95% confidence interval. FIQ, Fibromyalgia Impact Questionnaire.

Total costs correlated significantly and moderately with the degree of impact of the illness on the patient according to the FIQ (*r *= 0.282, *P *< 0.001) due to the fact that both indirect and health costs also were significantly correlated, though moderately, with the FIQ (*r *= 0.265 and 0.202, respectively; *P *< 0.001 and *P *= 0.004, respectively). However, when patients were analyzed with the responses to the FIQ grouped by deciles, a significant linear relationship (*P *< 0.001) of moderate intensity of the association was observed, both with total costs (*R*^2 ^= 0.55) and with indirect and health care costs (*R*^2 ^= 0.51 and 0.67, respectively) (Figure 1).

**Figure 2:**
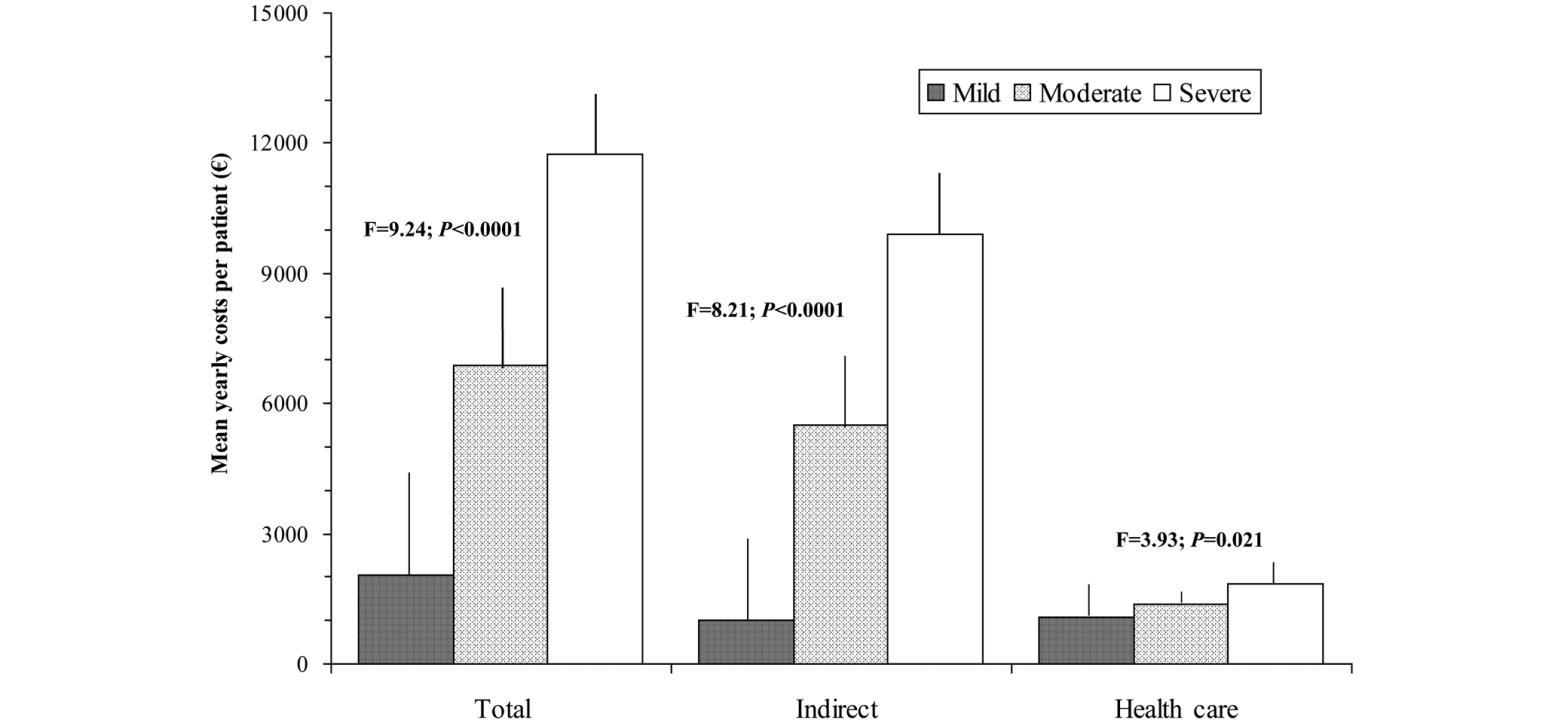
Annual mean costs of fibromyalgia patients according to severity of pain as assessed by the Brief Pain Inventory (BPI). The range of the BPI is from 0 to 10. The three levels of pain severity are mild (BPI <4; n = 9), moderate (BPI ≥ 4 to <7; n = 76), and severe (BPI ≥ 7; n = 115). Total, indirect, and health care costs are shown.

**Figure 3:**
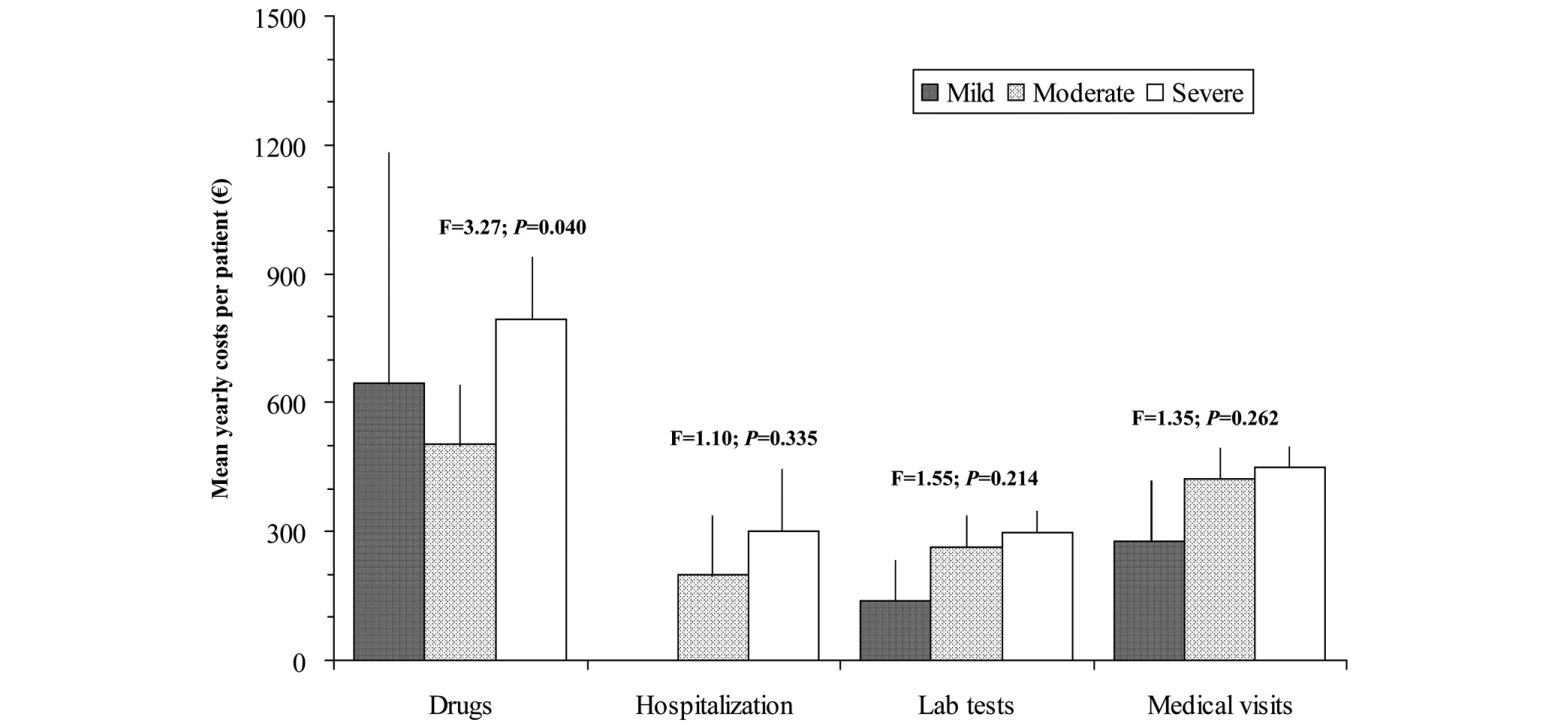
Annual mean costs of fibromyalgia patients according to severity of pain as assessed by the Brief Pain Inventory (BPI). The range of the BPI is from 0 to 10. The three levels of pain severity are mild (BPI <4; n = 9), moderate (BPI ≥ 4 to <7; n = 76), and severe (BPI ≥ 7; n = 115). Health care costs are split into components.

**Figure 4:**
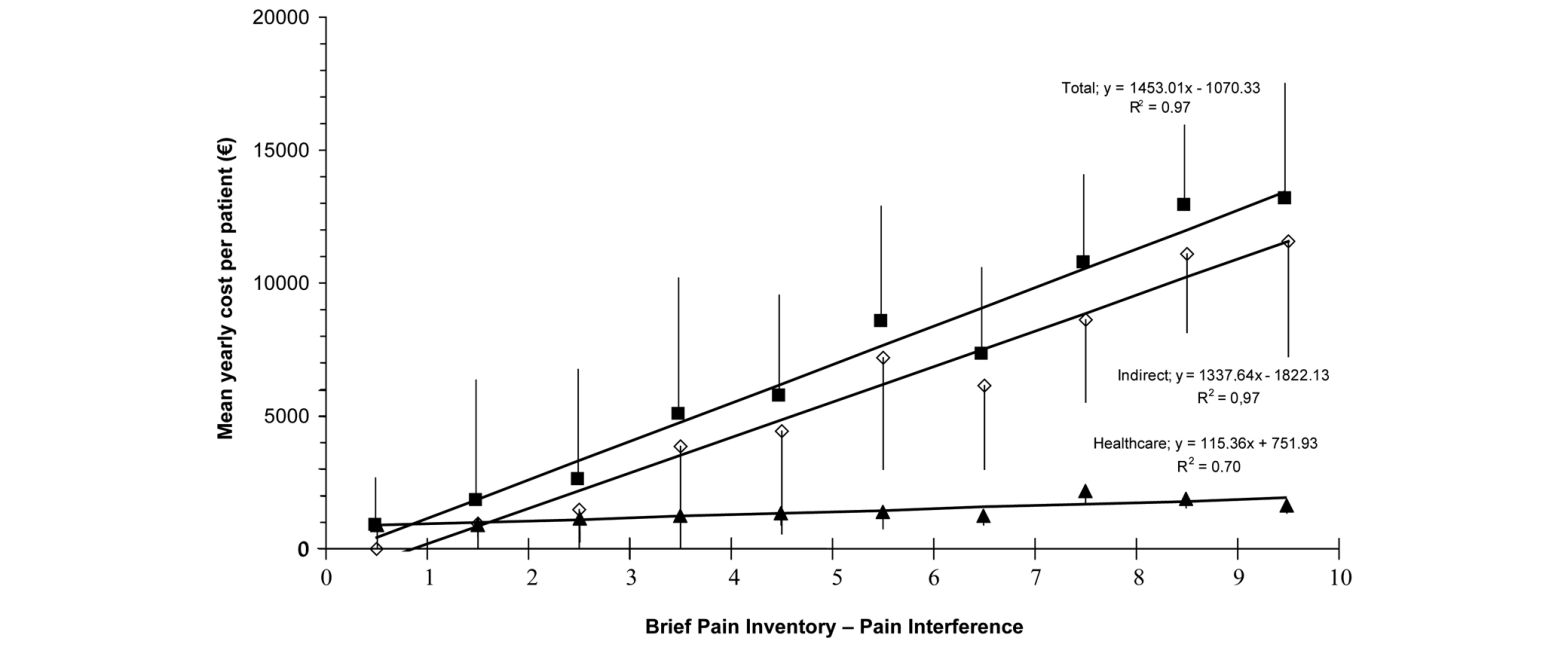
Ability of pain interference on patient's daily activities (BPI-I) to explain annual mean total costs. The range of the BPI-I is from 0 to 10. Explanatory ability assessed by linear regression models of level of interference on the BPI-I and corresponding costs of patients grouped by instrument intervals of pain interference are shown. Cost values are expressed as mean ± 95% confidence interval of subjects included in each interval. BPI-I, Brief Pain Inventory – Pain interference subscale.

**Figure 5:**
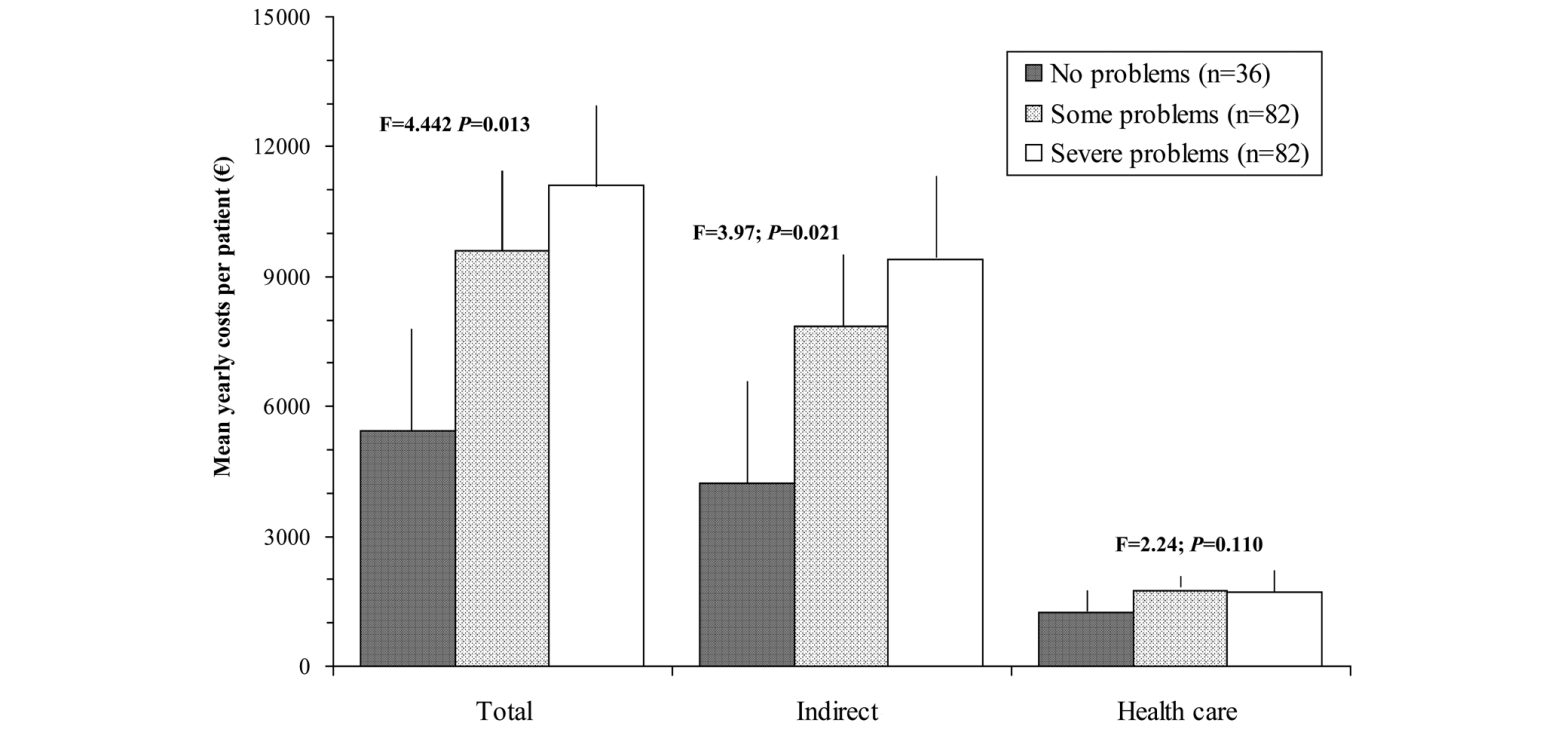
Annual mean total, indirect, and health care costs of fibromyalgia patients according to degree of problems in item 5 (anxiety/depression) of the five-item questionnaire on European quality of life (EQ-5D).

The intensity of the pain present in the patient, according to the BPI pain severity subscale, also correlated in a significant and moderate way to the total costs (*r *= 0.270, *P *< 0.001) and indirect costs and less with the direct health care costs (*r *= 0.180, *P *= 0.011), showing statistically significant differences (*P *< 0.05) when patients were grouped according to pain intensity (Figure 2). Direct health care costs and pain intensity due to FM were driven largely by pharmacological costs (Figure 3). However, pain interference in general activities and daily lives of the patients measured with the BPI interference subscale correlated in a significant way with total (*r *= 0.340, *P *< 0.001), indirect (*r *= 0.323, *P *< 0.001), and health care (*r *= 0.217, *P *= 0.002) costs. Figure 4 more clearly demonstrates the very significant linear relationship (*P *< 0.001) with *R*^2 ^association coefficients greater than or equal to 0.7 between the total, indirect, and health care costs and the degree of interference in daily activities caused by the pain as measured by the 11-point numeric rating scale. Finally, when patients were classified according to item 5 of the EQ-5D questionnaire (presence of symptoms of anxiety and/or depression) based on no problems, some problems, and extreme problems, statistically significant increases (*P *< 0.05) were observed in total and indirect costs, but not in health care costs with greater responses of problems (Figure 5).

## Discussion

This study determined the incremental costs of patients with FMS compared with a reference population using a local health provider that nonetheless may be representative of what occurs at many health care facilities in our current geographical environment. Incremental costs in relation to the reference population include the use of health care resources as well as employment or earning losses due to absenteeism or early retirement due to disability from regular employment before the age of 65, the theoretical retirement age in Spain. The analysis carried out shows that the most important component of the cost of illness corresponds to indirect costs or earnings losses, contributing approximately 81% in FM patients, compared with 71% in the reference population. Even though the relative data may not show the magnitude of the cost and burden of illness, patients with FMS have a mean incremental cost that is more than €5,000 greater annually than that of the reference population (nearly €4,400 due to earnings lost and more than €600 in direct health care resources), which (aside from being statistically significant) appears to us to be a considerable economic burden to society and is in line with that observed in pathologies classically considered first-order health problems (such as generalized anxiety or refractory epilepsy), once adjusted for currency and year (around €6,000) [32-35], and is approximately half of the cost observed in disorders such as vascular dementia and Alzheimer-type dementia, which are considered to be health disorders with a high economic burden on society [36,37].

Although losses of productivity due to early permanent disability before the age of 65 and leave from employment make up the most significant part of the cost, it should be mentioned that costs corresponding to health care resources were significantly higher in patients with FMS, with a mean annual excess greater than €600. Of these, 42% corresponds to the cost of pharmacological treatment, although this cost represents a mere 8% of the total. Also, the use of an annual average of 3.7 medications in patients with FMS (significantly higher than the 1.4 medications used by the reference population) gives us an idea of the difficulty of treating this syndrome and the degree of inefficiency of the current therapeutic armaments available for this health care problem. It is worth emphasizing that, with the exception of hospital costs, the other components of the health care cost (medical visits and supplementary tests, aside from those due to medications) were significantly higher in subjects with FMS and higher on the whole than that corresponding to drug costs. More specifically, patients with FMS had, in a 12-month period, six more medical visits on average than the general population, which agrees with the data from Hughes and colleagues [38], Wolfe and colleagues [39], and Robinson and colleagues [11], who also noted an increase in the use of medical services in FM patients which was much greater than that observed in the reference groups.

Analyses relating costs with measurements of self-perceived health have allowed us to prove the existing relationship between greater costs and the increased impact of FM on patient function (FIQ) and the intensity and interference of pain which these subjects have in their daily lives (BPI). The linear relationships found using the FIQ illustrated that an increase in the impact of FM by one decile (for example, going from the 6th percentile, 78.9 points on the FIQ, to the 7th, 81.5 points) increased total costs by approximately €865 annually. Regression analyses showed that the mean increment in costs mentioned above appeared with fewer scoring variations on the FIQ at the decile intervals of greater impact of the illness (above the fifth decile) than in the lower deciles. This means that, when the impact of disease reaches a severe level, lower increases of the impact are accompanied by higher cost increments. A similar phenomenon was observed using the BPI using the pain interference index. For example, an increase in the degree of pain interference by 1 point (0- to 10-point scale) is associated with a total annual cost increase of €1,453. Pain intensity measured with the BPI was significantly associated with total, indirect, and health care costs as pain intensity rose from mild to moderate to severe, although the increase in health care costs was driven primarily by increased medication costs. Finally, the presence of anxiety and/or depression symptoms (question 5 of EQ-5D) showed significantly higher total costs when the degree of anxiety/depression was severe, which was driven primarily by indirect costs.

We have not found any studies similar to ours in order to compare our findings with other health care areas related to FMS in our health care environment. However, when we compare our results to those published by other authors in other contexts, some discrepancies are observed. For example, our results differ greatly from those of a study by Berger and colleagues [14], which is also on the cost of FMS and performed using an administrative database in the US. That study shows that, after currency conversion and adjustment for the year of the study, costs are similar in amount to ours, but only reflecting direct health care costs. The explanation for this difference could be that these are very different health care systems with very disparate fees and prices and even with distinct assistance protocols. However, results more similar to ours were observed in other studies, both in the US and in Canada [15,39], and above all, it was shown that comorbid depression significantly increased total costs [40]. These results were more coherent with our findings in that the presence of severe anxiety and/or depression problems existing alongside FMS significantly increased the total costs of the illness (in particular, indirect costs).

This work has allowed us also to explore the burden of the illness relative to the concurrent presence of other health disorders along with FMS. First, the prevalence of FMS in our population was slightly less than the national total, 1.7% versus 2.4%, with clear higher frequency in females [5]. What is remarkably noticeable is the high number of comorbid disorders that are significantly more frequent in patients with FMS than in the general reference population, as was previously indicated [41-43]. Thus, not only is there greater psychiatric comorbidity [44,45], but in the recent OMERACT (Outcome Measures in Rheumatoid Arthritis Clinical Trials) Delphi study [41], we also observed a greater presence of pain syndromes (20.5% versus 5.5%, 2.9 times more frequent), a greater prevalence of obesity/weight gain (41%), lipidic metabolism disorders (total cholesterol, low-density lipoprotein cholesterol, and triglycerides significantly higher than in the general population), migraines, and digestive disorders such as gastroesophageal reflux, gastritis, or irritable bowel, which are health disorders (already seen in other studies) that proved to be more prevalent in patients with FMS than in the general population, as reflected by the type of medication and greater consumption of medications by these patients [4,5,7,11,14,15,38,39,41].

This study has some limitations that must be pointed out. First are those which are relative to its cross-sectional and retrospective design, though usual for this type of study, which enable the potential underestimation of some costs relative to resources consumed outside of the BSA system or those not accurately recorded in the computer system of this health care provider. Given the quality of system data collection, subject to periodic monitoring for quality and motivation, and the infrequency of patient referrals to other health care systems, this potential underestimation of the costs is slight. However, we should recognize that costs have been underestimated with regard to 'out-of-pocket expenses', meaning those costs that are borne solely by the patient, such as special diets, modifications to their residences, or non-pharmacological treatments (massages, hydrotherapy, acupuncture, and so on) and that are not usually included in the BSA database and, as such, have not been taken into account in this study. On the other hand, this study has not provided a cost for disability days from any activity either in the retired population such as domestic chores (preparing food, ironing, and so on) or in leisure activities, as indicated by some health care economics experts. Nonetheless, given the academic controversy over the applicability of a monetary value to these costs, we decided not to include them in this study. Another possible limitation refers to the system followed to code diseases, the ICD-10v10. Even the ICD-10 coding replaced the version 9 to avoid misclassification detected in the oldest versions; we cannot guarantee completely that a proportion of subjects in the BSA database could not be correctly classified with an FMS diagnosis. Or even the opposite could have happened: patients without FMS could have received a code for FMS.

Another potential limitation refers to the administration of the instruments of self-perceived health (FIQ, EQ-5D, and BPI) over the telephone and doubts that this could produce reliable responses from patients. Although it is true that administration was performed without computer support (computer-assisted telephone interviews or a similar method), these instruments nonetheless have been used quite frequently in mail surveys sent to homes and are instruments that are easy to understand and well used in daily clinical practice, which is why we believe that the errors in the degree of accuracy should be minor.

## Conclusions

Taking into account the above-mentioned limitations, this study has determined the incremental costs to society for patients with FM in relation to the reference population and has related this to the level of pain, impact, and interference of the illness on the patients' daily lives. Our analysis shows that the cost of illness of this syndrome is substantial, particularly in relation to employment losses, which make up around four fifths of the illness cost, which should encourage clinics, health decision-makers, and health care authorities to set in motion some social-health measures that would contribute to the reduction of these alarming costs. Lastly, but by no means less importantly, the high use of health care resources relative to the reference population in the number of all types of medical visits as well as supplementary tests and drugs (which shows the level of inability and inefficiency of the health care resources currently available to clinics to alleviate the impact of this syndrome on the quality of life for these patients) should be indicated and should also motivate reflection upon this matter and a search for new solutions in the diagnostic and therapeutic management of FMS patients.

## Abbreviations

ACR: American College of Rheumatology; ANCOVA: analysis of covariance; BPI: Brief Pain Inventory; BSA: Badalona Serveis Assistencials S.A.; EQ-5D: five-item questionnaire on European quality of life; FIQ: Fibromyalgia Impact Questionnaire; FM: fibromyalgia; FMS: fibromyalgia syndrome; GERD: gasroesophageal reflux disease; ICD-10: International Statistical Classification of Diseases and Related Health Problems, 10th revision; PC: primary care; RUB, resource utilization band.

## Competing interests

JR is employed by Pfizer España (Madrid, Spain), which provided a grant that partially funded this study. The other authors declare that they have no competing interests.

## Authors' contributions

AS-M and JR participated in the design and analysis of this study and in the writing of the manuscript. RN participated in the preparation of the manuscript and in the literature review and extraction. MB participated in the interpretation of data and in the preparation of the manuscript. AM, RL, SV, and CV participated in subject evaluation and determination of eligibility for this study. All authors read and approved the final manuscript.
